# Cloning and characterization of feline islet glucokinase

**DOI:** 10.1186/1746-6148-10-130

**Published:** 2014-06-10

**Authors:** Sara Lindbloom-Hawley, Michelle LeCluyse, Vanessa Vandersande, Gerald Henry Lushington, Thomas Schermerhorn

**Affiliations:** 1 College of Veterinary Medicine, Department of Clinical Sciences, Kansas State University, 1800 Denison Ave, Manhattan, KS 66506-5606, USA; 2 LiS Consulting, 2933 Lankford Dr, Lawrence, KS 66046, USA

**Keywords:** Glucose sensor, Pancreas, Carbohydrate metabolism, Hexokinase, Gene expression

## Abstract

**Background:**

Glucokinase (GK) is a metabolic enzyme encoded by the *GCK* gene and expressed in glucose-sensitive tissues, principally pancreatic islets cell and hepatocytes. The GK protein acts in pancreatic islets as a “glucose sensor” that couples fluctuations in the blood glucose concentration to changes in cellular function and insulin secretion. *GCK* and GK have proposed importance in the development and progression of diabetes mellitus and are potential therapeutic targets for diabetes treatment. The study was undertaken to determine the nucleotide sequence of feline pancreatic GK cDNA, predict the amino acid sequence and structure of the feline GK protein, and perform comparative bioinformatic analysis of feline cDNA and protein. Routine PCR techniques were used with cDNA from feline pancreas. Clones were assembled to obtain the full length cDNA. Protein prediction and modeling were performed using bioinformatic tools.

**Results:**

Full-length feline pancreatic GK cDNA contains a 1398 nucleotide coding sequence with high identity to other pancreatic GK cDNAs. The deduced 465 amino acid feline protein has 15 amino acid substitutions not found in other mammalian GK proteins but maintains high structural homology with human GK. Feline pancreatic GK is highly conserved at nucleotide and protein levels. Residues crucial for substrate binding and catalysis are completely conserved in the feline protein.

**Conclusion:**

Molecular analysis predicts that feline pancreatic GK functions similarly to other mammalian GK proteins.

## Background

Glucokinase (GK) is a metabolic enzyme encoded by the *GCK* gene and expressed in glucose-sensitive tissues, principally pancreatic islets cell and hepatocytes. Pancreatic and hepatic GK enzymes are isoforms that differ slightly in the N-terminus amino acid sequence [1]. The isoforms arise from tissue-specific mRNAs that are encoded by a single *GCK* gene under control of separate promoters [1]. In pancreas and liver, the GK protein functions as a “glucose sensor” that couples fluctuations in the blood glucose concentration to changes in cellular function. Biochemical analysis has determined that the metabolic control strength of pancreatic GK for glucose metabolism is near unity, a finding supported by results of gene expression studies that shows that the *GCK* gene exerts a dose dependent effect on glucose metabolism [2].

Knowledge about feline GK and its metabolic role is incomplete. The domestic cat (*Felis catus*) is an obligate carnivore and must have evolved specialized mechanisms suited for consumption of a diet containing large amounts of protein but relatively little carbohydrate (reviewed by [3]). Several early studies reported reduced or absent activity of the GK enzyme and its regulatory protein (GKRP) in feline liver when compared with other species [4,5]. Recent studies have shown that the feline liver does not express mRNA for GK [5] or GKRP [6], which suggests that reduced expression of genes involved in glucose-sensing is responsible for the low GK enzyme activity observed in early studies. Although GK mRNA expression has been documented in the feline pancreas [5,7], expression and activity of the feline pancreatic GK isoform has not been studied in depth. Given the central role of pancreatic GK as glucose-sensor for insulin-producing islet cells and the requirement for GK in normal glucose metabolism and insulin secretion, detailed knowledge about GK expression and function may improve understanding of feline carbohydrate metabolism.

The objective of the current study was to characterize the feline pancreatic GK isoform and compare the feline isoform with GK from non-carnivorous mammals. Study aims were to determine the nucleotide sequence of pancreatic GCK cDNA, predict the amino acid sequence and structure of the feline GK protein, and perform bioinformatic analysis of the feline cDNA and protein.

## Results and Discussion

### Feline GK cDNA coding sequence and alignments

**Figure 1:**
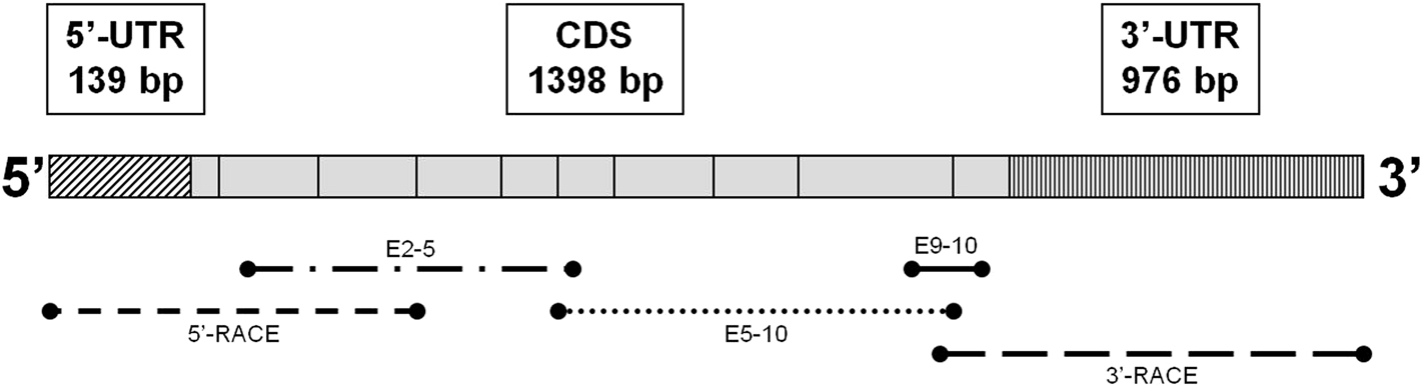
**Schematic representation of the feline pancreatic GK cDNA.** 5’ and 3’ untranslated regions (UTR) are represented by hatched blocks. The coding region is divided into 10 exons, which are represented by gray boxes; the relative size of each box reflects exon length. The position of DNA clones produced by each primer set used in PCR reactions is shown relative to the assembled cDNA sequence (Genbank accession EF121813).

Structural features of the complete feline pancreatic GK cDNA are shown in Figure 1. Alignment of DNA clones yielded a 2513 bp cDNA sequence with 9 putative ORFs. An ORF beginning at base 140 of the cDNA sequence spanned 1398 bp and represented the complete feline GK CDS. Nucleotide identities between the feline CDS and sequences from human, chimpanzee, rat, and mouse were 92%, 92%, 87%, and 88%, respectively. Using the known exonic structure of human GK mRNA (Genbank accession NM_000162), it was determined that feline GK contains 10 exons. RACE cloning yielded sequence information for putative 5’and 3’ UTRs contained in the feline GK mRNA. The 5’-UTR contained a 139 bp sequence located immediately upstream to the ORF that encodes the 1398 bp CDS. The 976 bp immediately following the CDS stop codon comprises the 3’-UTR.

Additional alignments were carried out using the fGK cDNA sequence and the feline genomic sequence (Felis_catus-6.2 reference assembly). Since the linear cDNA contains only exon information the alignment was limited to coding regions of the feline GK gene. The 5’-UTR and exon 1 of fGK cDNA had 100% identity with a short 184 nt genomic sequence located approximately 32 kb upstream from the start of exon 2. Overall, nt identity between fGK exons 2–10 and the 3’-UTR, excluding intervening intron sequences, was 99.8%. Three A/G nucleotide differences were detected in the 3’-UTR sequences. A T/C change at position 119 in exon 9 was the only non-identity in a coding region.

### Predicted feline pancreatic GK protein sequence, alignments and bioinformatic analysis

**Figure 2:**
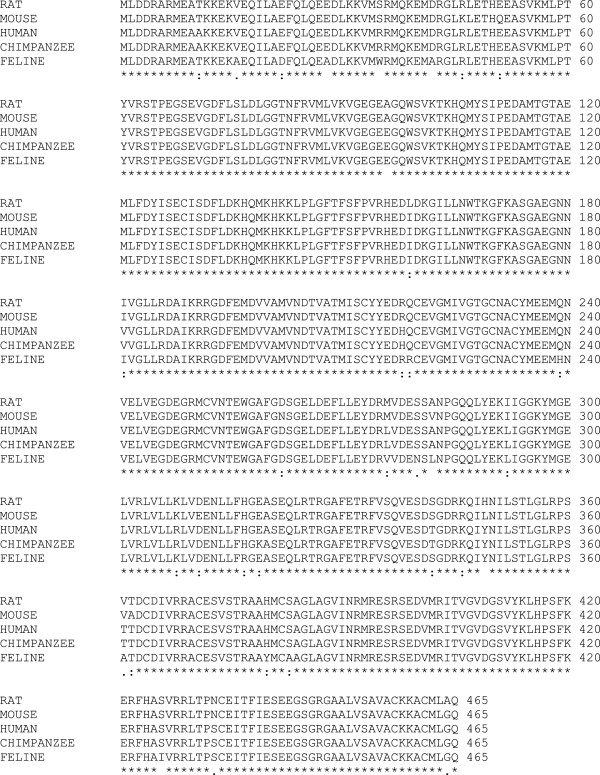
**Alignment of feline GCK with other mammalian GCK sequences.** *= Identity at this position in all aligned sequences. : = Conserved substitutions at this position in one or more aligned sequences. . = Semi-conserved substitutions at this position in one or more aligned sequences <space> = non-identity or nonconserved substitution at this position in one or more aligned sequences.

The feline pancreatic GK protein deduced from the 1398 bp CDS found in the cDNA is 465 amino acids in length, and has a calculated molecular weight of 52215.67 Daltons or approximately 52 kD (kiloDaltons). Amino acid sequence identities with human, chimpanzee, rat and mouse sequences ranged from 89-94% (Figure 2).

Structural and mutational analyses of human GK have identified residues 78–85, 151, 169, 205, 225–228, 295–296, 331–333, 336, and 410–416 as important in ATP binding [8] and residues 151–153, 166–169, 204–206, 225–231, 254–258, 287, and 290 as important for glucose binding [8,9]. The amino acids involved in ATP- and glucose-binding in human GK are completely conserved in feline GK (Figure 2). Amino acid residues (Lys 58, Asn 204, Leu 309, Leu 314, Asn 350, and Leu 355) previously found to mediate GK binding to its regulatory protein [10] are also completely conserved in feline pancreatic GK (Figure 2). Feline pancreatic GK contains a leucine-rich 11-aa sequence (^300^ELVRLVLLKLV^310^) near the C-terminus of the protein that is likely to act as a nuclear export signal (NES) (Figure 2).

**Figure 3:**
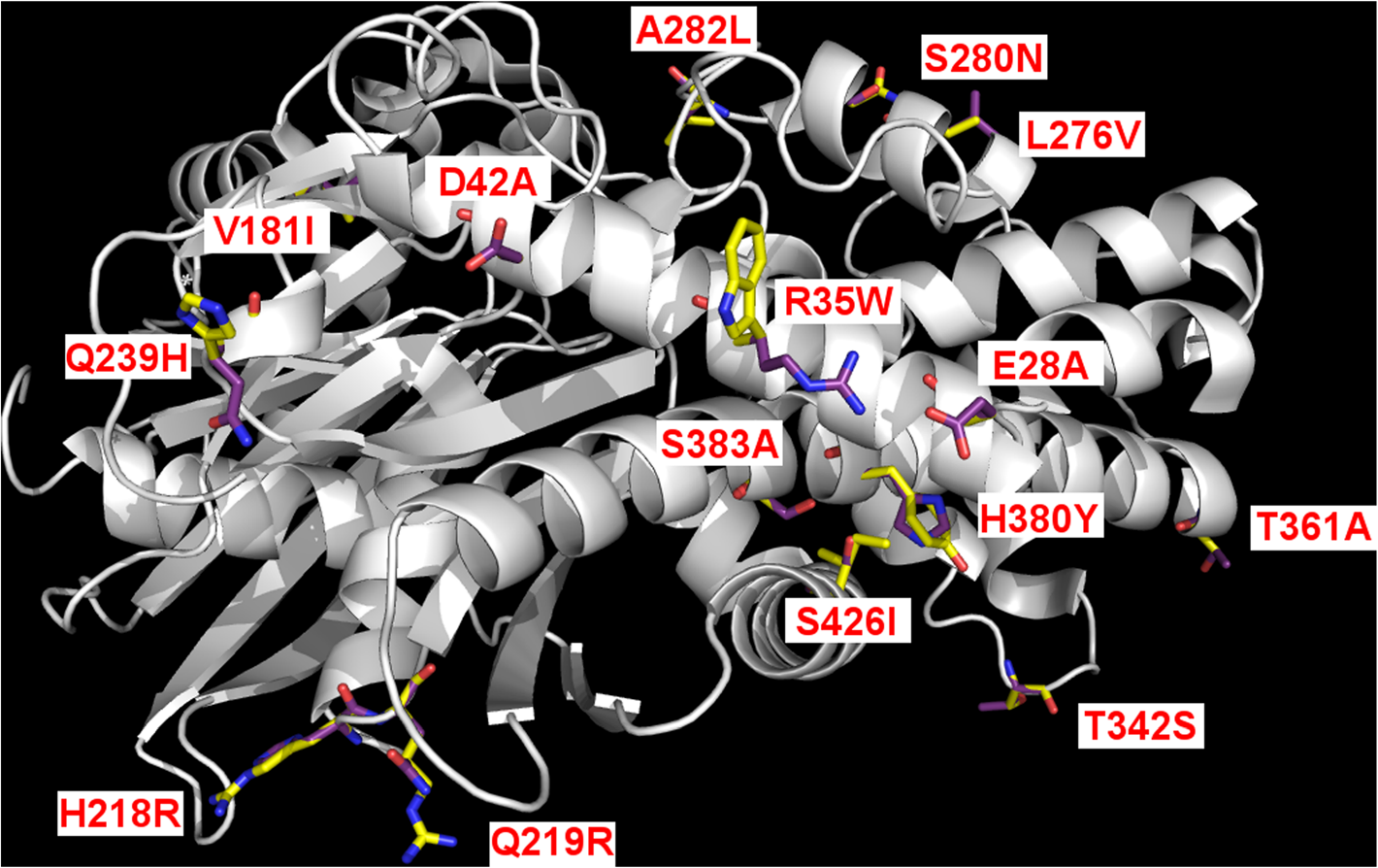
**Amino acid comparison between fGK and human GK**. The location of amino acid differences within the feline and human GK proteins are shown. For each position where the sequence differs, the label shows the single letter code for the human aa, the position in the sequence, and the corresponding feline aa. Amino acid residues are shown in stick form with feline residues shown in yellow and corresponding human residues in purple.

Fifteen amino acid residues found in feline GK are not found in corresponding positions in human, mouse, rat or chimpanzee GK (Table 1; Figure 3). Of these, 10 are considered moderately or highly conserved substitutions and unlikely to affect protein structure or function. The remaining 5 residues (alanine^28^, tryptophan^35^, alanine^42^, leucine^282^, isoleucine^426^) are non-conserved substitutions. Three non-conserved residues (alanine^27^, tryptophan^35^, alanine^42^) are located near the N-terminus and away from residues involved in ATP or glucose binding and catalysis. One of the non-conserved residues (leucine^282^) occurs at a site of amino acid variability in other species (alanine in rat, human and chimpanzee; valine in mouse). The fifth non-conserved residue (isoleucine^426^) is found near the protein C-terminus, where no crucial residues have been identified. Phylogenic analysis of several mammalian GK proteins shows the feline GK protein is closer to primate GKs than to rodent GKs.

**Table 1:** Conserved and non-conserved amino acid substitutions in feline pancreatic GK

**Position**	**Amino acid residue**	
	**Feline GK**	**Mammalian GKs**^**‡**^
16	Ala	Val
22	Asp	Glu
**28**	**Ala**	**Glu**
**35**	**Trp**	**Ser (R) or Arg (P)**
**42**	**Ala**	**Asp**
219	Arg	Gln
239	His	Gln
276	Val	Met (R) or Leu (P)
280	Asn	Ser
**282**	**Leu**	**Ala**^**†**^
317	Arg	His
361	Ala	Val (R) or Thr (P)
380	Tyr	His
383	Ala	Ser
**426**	**Ile**	**Ser**

^‡^Alignments performed using human, chimpanzee, rat, and mouse GKs.

Conserved substitutions are shown in normal text.

Non-conserved substitutions are shown in **bold text**.

R = rodents (rat and mouse).

P = primates (human and chimpanzee).

^†^This residue is Ala in rat, human, and chimpanzee; Val in mouse.

The 465 aa feline GK protein sequence predicted from pancreatic cDNA is longer than the 450 aa GK protein (Ensembl Protein ID: ENSFCAP00000013323) coded by a 1353 bp GK cDNA (Ensembl Transcript ID: ENSFCAT00000014365) predicted from analysis of feline genomic DNA. The discrepancy in protein size is due to the presence of 15 additional amino acids on the N-terminal of the fGK protein. Direct comparison revealed three amino acid differences between the two feline GK sequences. The fGK protein differs from the Ensembl sequence at aa 15 (Lys vs. Gln) and 380 (Tyr vs. His). In addition, Val 99 present in fGK corresponds with a gap in the alignment with the Ensembl sequence. The predicted size and sequence of fGK is more compatible with other mammalian GK protein sequences than the Ensembl sequence, all of which are 465 aa in length and contain Lys and Val at the 15 and 91 positions. However, Tyr 380 in fGCK is not found in other mammalian sequences, which like the Ensembl feline protein have a His residue at this location.

### GK structural model

**Figure 4:**
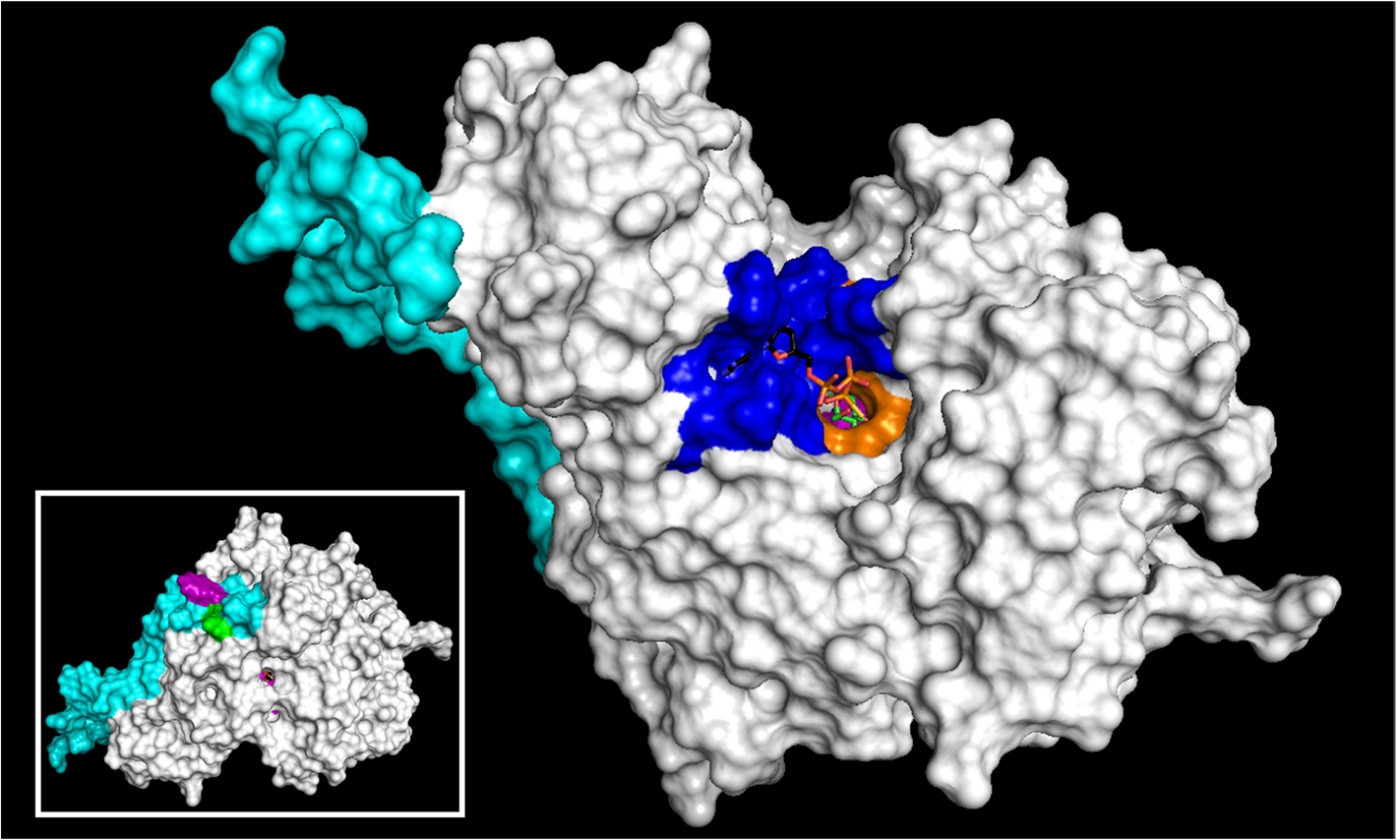
**Homology model of feline pancreatic GK protein**. The protein is shown bound to molecules of ATP and glucose with the binding site and catalytic pocket facing outward. ATP (not shown) binds deeper in the pocket than glucose (shown in green). The residues involved in ATP binding (dark blue) are completely conserved in the feline GK protein, as are the residues involved in glucose binding (orange). Some residues are involved in both ATP and glucose binding (pink). *Inset* – The face of the GK molecule opposite the binding pocket is shown. The N-terminal portion of the GK molecule (light blue) contains three of the five non-conserved amino acid substitutions found in feline pancreatic GK. Feline GK has trp-arg (shown in purple and green, respectively) at positions 35 and 36 while other species have arg-arg or ser-arg in these same positions. As a result of the presence of trp at position 35 and ala at position 28 (another non-conserved feline variant) feline GK lacks a salt bridge that is present in human GK.

**Figure 5:**
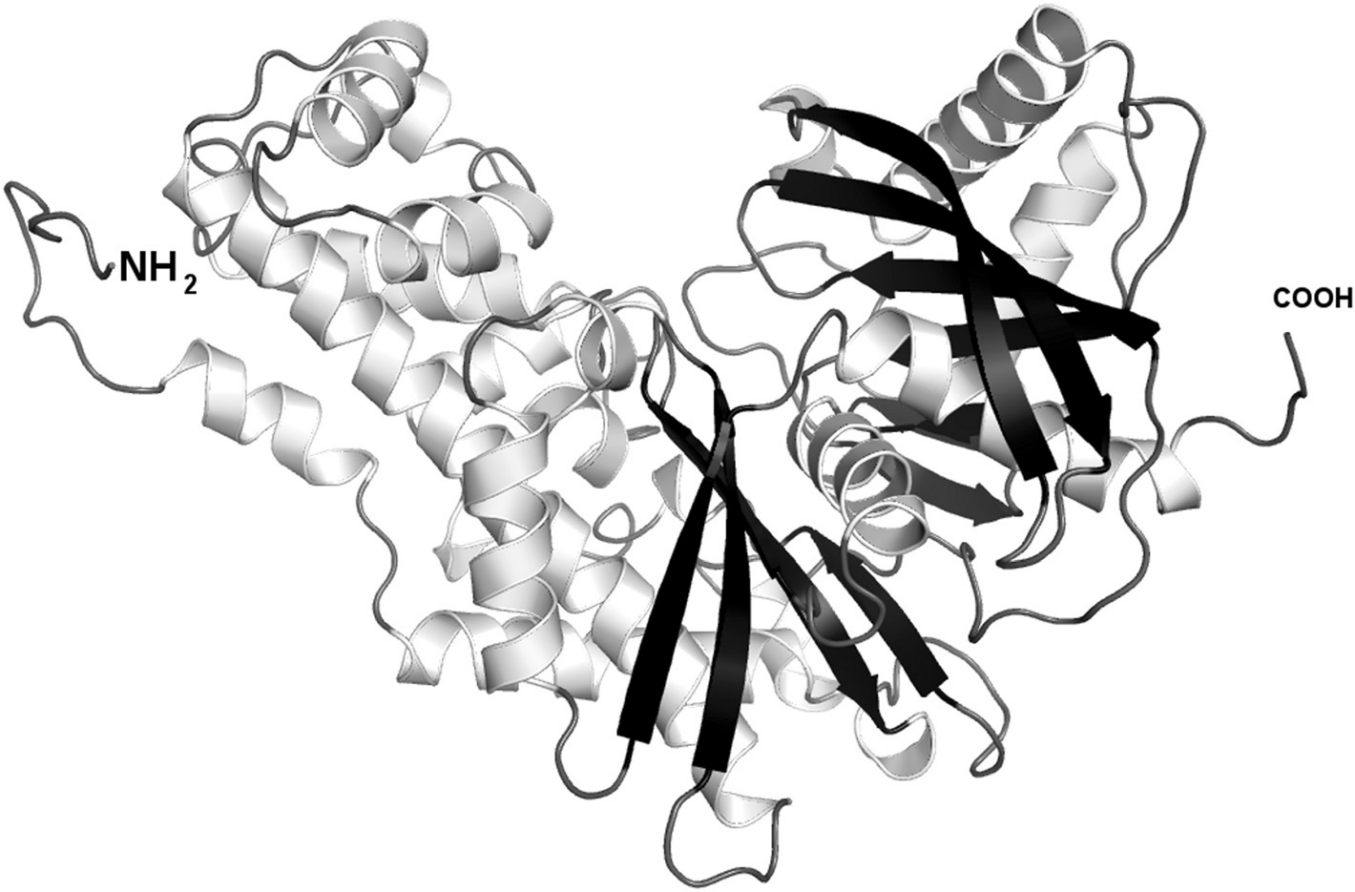
**Secondary structure of feline pancreatic GK showing alpha helices (light coils) and beta sheets (dark arrows).** There are two globular domains separated by a hinge region that allows the globular domains to open and close during substrate binding and release. The cleft that is formed contains the sites of glucose and ATP binding.

Homology modeling of the predicted feline GK protein (Figure 4) demonstrated high structural homology with the crystallographic structure of human glucokinase determined by Kamata et al. [11]. Secondary structure analysis of feline GK reveals the protein has two globular domains separated by a hinge region (Figure 5). The cleft formed between the globular regions contains the residues involved in glucose and ATP binding. The predicted feline GK structure would permit the globular domains to open and close during substrate binding and is consistent with a currently proposed mechanistic model of GK function [12]. The non-conserved amino acid variants present in feline GK conveyed no appreciable structural consequences. Three of the non-conserved residues lie within a N-terminus 43-aa stretch which forms part of the surface of the GK molecule that is opposite the surface that forms the substrate binding cleft and catalytic region. The feline GK sequence at positions 35 and 36 is trp-arg while other species have arg-arg or ser-arg in these same positions. As a result of the presence of trp-35 and ala-28 (another non-conserved feline variant) feline GK lacks a salt bridge that is present in human GK [11]. Furthermore, trp-35 is fully solvent exposed in the model, which may make this portion of the molecule more lipophilic.

## Discussion

The results provide the initial description of the feline pancreatic GK mRNA and protein, including the complete nucleotide sequence, amino acid sequence, and molecular structure. The 2.5 kb feline cDNA contains a 1398 CDS that has a high nucleotide identity with pancreatic GK cDNA sequences isolated from other mammals. The feline cDNA encodes a 465 aa protein that has a high degree of sequence and structural homology with mammalian pancreatic GK proteins.

The coding region of feline cDNA has a high overall nucleotide identity with GK mRNAs isolated from pancreases of other mammalian species. Percent (%) nucleotide identity is highest with pancreatic GK cDNAs from primates (>90%) and slightly less with rodent pancreatic GK cDNAs. The latter finding is consistent with a trend observed in other feline genes involved in carbohydrate metabolism, such as *G6PC* (glucose-6-phosphatase catalytic subunit) [13] and *KHK* (ketohexokinase) [14], which have shown greater nucleotide identity with primate and canine sequences than with rodent species. The ten putative exons identified in feline GK have homology with established exon sequences of human pancreatic GK. Exon-exon borders in the feline sequence are identical to those in human GK and feline exons are similar in length to corresponding human exons. Based on extensive homology with human exon 1A, which is expressed in pancreatic endocrine cells but not in hepatocytes, exon 1 of feline pancreatic GK can be designated as exon 1A. GK mRNAs containing alternative exon 1 sequences have been isolated from rodent and human tissues [15-17]. Alternatively spliced GK mRNA isoforms are typically detected in liver [15,17]. Islet glucokinase mRNA is present as a single transcript produced via activation of the neuroendocrine (pancreatic) promoter. Consistent with findings from other mammals, the feline pancreas was found to produce a single GK cDNA with homology to mammalian pancreatic GK and no alternatively spliced pancreatic isoforms were detected.

Although GK activity has not been demonstrated in the feline pancreas, pancreatic GK activity is essential for normal glucose homeostasis in humans and rodents and a similar role is almost certainly true in cats. Comparative analysis of feline GK at the protein level showed amino acid identities to be slightly higher than at the nucleotide level but the comparisons followed the same general pattern across the species examined (Figure 2). Feline GK has five non-conservative amino acid substitutions that are unique to the feline protein (i.e. these residues do not appear at the same location in GK from other mammalian species examined). Non-conserved amino acid substitutions are of interest because the introduction of unique amino acids into GK during feline evolution has the potential to impact global protein function through any of a number of molecular mechanisms, as observed in human GK mutations [18,19]. Among the amino acid residues unique to feline GK, the most interesting for its possible functional consequences is Trp35. Unlike the cat, rat, mouse, human, and chimpanzee all have an Arg residue in this position. Interestingly, an arg→trp mutation at position 36 (adjacent to the feline substitution) in *GCK* was detected in a human patient with GCK-MODY (also called MODY2), a monogenetic type of diabetes [20]. *In vitro* studies that examined the effects of the R36W mutation on the GK enzyme found that the kinetic characteristics and thermal stability of the R36W mutant human GK did not differ from wild type human GK [19]. However, the R36W mutation is located in a region of the GK protein that may be important for interaction with GK regulators and proteins involved in cellular GK localization [19]. The positively-charged arginine residue at position 35 (arg 35) forms a salt bridge with glu 28 in human GK but the trp 35 substitution in feline GK abolishes the salt bridge with glu 28. In addition, trp 35 is fully solvent exposed in the structural model of feline GK, which would suggest this region is more lipophilic than the same region on human GK. Both of these predicted structural changes could potentially impact feline GK protein dynamics, perhaps by influencing protein-protein interactions or protein stability, as postulated for the R36W mutation of human GK.

When GK is expressed in mammalian liver cells its cellular localization is determined by its interactions with GKRP. GKRP is not expressed in pancreatic endocrine cells but there is evidence that protein interactions are involved in GK localization in beta cells. The specific GK residues that mediate binding with GKRP are located in the small domain and hinge region in the folded protein and partly overlap with the glucose-binding domain [10]. The amino acids involved in interaction with GKRP are fully conserved in feline GK but since feline liver does not express GKRP [6], retention of these residues in the feline protein likely reflects the high degree of overall amino acid conservation in the GK protein and the dual role of some of these residues in GKRP and glucose binding. GK interaction with GKRP is needed for entry into the nucleus [21] but GKRP is not required for GK to exit the nucleus. Nuclear export signal (NES) sequences enriched in leucine residues are a characteristic of proteins, like GK, that can traverse the nuclear membrane. A leucine-rich NES encompassing positions 300–310 has been defined in rat GK and similar sequences are found in human, mouse, and chimpanzee GKs, including conservation of all lysine residues. In feline GK, the putative NES sequence is identical to that found in rat GK [21].

Direct determination of cDNA sequences for transcripts expressed in specific tissues can be difficult and time consuming but the approach has several advantages over methods that employ gene mining software to identify putative genes and predict cDNAs from a genomic DNA template. Direct cDNA sequencing is a particularly apt method for investigation of the GK gene, which is controlled by multiple promoters and exhibits tissue specific mRNA expression. The feline cDNA and protein we report here contain structural and sequence features that differ from archived feline cDNA and protein sequences in the Ensembl data base. In particular, the fGK cDNA and corresponding GK protein sequences we determined are longer than those determined by primary sequencing of feline genomic DNA. Comparison of the protein sequences revealed that the Emsembl protein lacks 15 aa that are present in the 465 aa fGK predicted from pancreatic cDNA. These 15 aa comprise the exon (pancreatic exon 1) specifically encoded by mRNA produced by activation of the GK pancreatic promoter. No sequence corresponding to the pancreatic exon 1 is contained in the Emsemble cDNA or protein sequences. In human GK, the pancreatic exon 1 is located near the pancreatic (neuroendocrine) promoter, which is far upstream of the remainder of the coding region (exons 2–10) common to pancreatic and hepatic GK isoforms. A similar situation is likely to occur in the feline GK gene since the genomic location of the 184 nt sequence homologous to the feline GK 5’-UTR and pancreatic exon 1 is approximately 30 kb upstream of exon 2. Inability of the predictive software to recognize a short start sequence so distant from the next exon likely accounts for the size discrepancy between our reported cDNA and protein and the Ensembl sequences.

There are few GK sequences from individual cats available for comparison. Although overall sequence identity between the fGK cDNA and the putative GK gene contained in the feline genome is very high, several nucleotide differences were noted. The majority of the differences occurred in the UTR and do not affect protein sequence but one possible non-synonymous SNP was identified. The T/C SNP occurs within exon 9 and produces either a Tyr (fGK protein) or a His (Ensembl feline GK protein). The Tyr at position 380 is unique to feline GK as a His residue occurs in that position in most mammalian GK sequences (Figure 2). This was considered a non-synonymous (non-conserved) difference because Tyr is an uncharged, aromatic aa and His is a charged aa. Modeling the fGK protein with Tyr^380^ had no appreciable effects on the protein structure but functional studies are required to fully assess the impact of this possible SNP on the GK protein.

## Conclusions

In summary, the pancreatic isoform of feline GK is highly conserved at the mRNA and protein levels. All residues crucial for substrate recognition and binding, protein interactions, and localization are completely conserved in the feline protein. Several amino acid substitutions unique to the feline protein were identified but do not significantly impact GK structure in the protein model. However, the impact of each substitution on GK structure and function was not directly determined. Based on the molecular analysis performed, it is likely that feline pancreatic GK functions in a manner similar to other mammalian pancreatic GK proteins.

## Methods

### Tissue source

Pancreatic tissue was obtained at necropsy from a non-diabetic domestic cat that had died of natural causes. Because no live animals were involved, the studies did not require approval of the Institutional Animal Care and Use Committee. All procedures and protocols were in compliance with institutional guidelines for research involving biological materials.

### Overview of the cloning strategy

A series of primers were designed against conserved regions in published sequences of mammalian GK cDNAs and used to amplify regions of feline pancreatic cDNA. The PCR products obtained were sequenced and new primers designed using feline specific DNA sequences. A series of primer sets (Table 2) generated a series of overlapping clones that when assembled comprised the complete feline GK cDNA.

**Table 2:** Primer sets used in PCR reactions

**Primer set**	**Sense**	**Anti-sense**
E2-5	5’-GCAGGAGGCCGACTTGAAGA-3’	5’-CCATCAAACGGAGAGGGGACTT-3’
E5-10	5’-TCGTGGGGCTCCTACGTGAT-3’	5’-GCACCCCAGCTTCAAGG-3’
E9-10	5’-GCCGCAGCGAGGACGT-3’	5’-GTGCGCAGGCTGACGCC-3’
3’-RACE	5’-GGGCGTGGACGGCTCTGTGTACAAACTGCA-3’	Provided by kit manufacturer
5’-RACE	Provided by kit manufacturer	5’-CCATGACGGGTACTGCTGAG-3’

### Reverse transcriptase-PCR (RT-PCR)

Total RNA was isolated from feline pancreas (RNeasy Micro Kit, Qiagen) and reverse transcribed into cDNA (Omniscript Reverse Transcription Kit, Qiagen). PCR was carried out with the Expanded High Fidelity PCR System (Roche Applied Science) and a TGradient Thermocycler (Biometra) using various primers sets (Table 2) and feline pancreatic cDNA as the template. Thermal cycling parameters were 94°C for 5 min, followed by 34 cycles of amplifications at 94°C for 30 s, annealing for 30 s, 72°C for 45 s, and 72°C for 7 min as the final elongation step. The annealing temperature used varied depending on the primers used.

### Cloning and sequencing

Gel electrophoresis on a 1% agarose gel was used to identify PCR products of the correct size. Products were isolated from the agarose (DNA Gel Extraction Kit, Millipore Corporation), and ligated into a vector containing the gene for ampicillin resistance (pGEM-T Easy Vector, Promega). The vector containing the PCR product insert was used to transform DH5α cells, which were spread on agar plates (100 μg/mL ampicillin) and incubated overnight at 37°C. Individual colonies were used to inoculate 5 mL cultures, which were allowed to incubate overnight at 37°C while shaking. Plasmids were then isolated (QIAprep Spin Miniprep Kit, Qiagen) from the DH5α cells. Presence of the insert was determined by EcoR1 restriction digest, and plasmids containing the correct insert were sequenced using the T7 promotor primer at the University of California at Davis Sequencing Facility (ABI 3730 DNA Sequencer).

### RACE cloning

Feline-specific primers were designed to be used for RACE cloning using the GeneRacer Kit with SuperScrip III Reverse Transcriptase (Invitrogen). Total RNA was isolated from feline pancreas using the RNeasy Micro Kit (Qiagen). The cap structure was removed from mRNAs and the GeneRacer RNA Oligos were ligated to the 5’ ends. Messenger RNAs were then reverse transcribed into cDNA using SuperScript III Reverse Transcriptase and the GeneRace Oligo dT Primer. For amplification of the 5’ and 3’ ends of the GK cDNA, PCR was performed with the GeneRacer 5’ and 3’ RACE kits according to manufacturer’s instructions using specific 5’ and 3’ end primers provided by the manufacturer and gene-specific primers designed using the feline GK sequence (Table 2). PCR amplification was done by touchdown PCR using the Expanded High Fidelity PCR System (Roche Applied Science) and the TGradient Thermocycler (Biometra).

Gel electrophoresis on a 1% agarose gel was used to identify products of the correct size. Products were isolated from the agarose (DNA Gel Extraction Kit, Millipore Corporation), and ligated into the TOPO Cloning Vector using the TOPO TA Cloning Kit for Sequencing (Invitrogen). TOPO vector was used to transform One Shot TOP10 Chemically Competent Cells (Invitrogen), which were spread on agar plates (100 μg/mL ampicillin) and incubated overnight at 37°C. Individual colonies were used to inoculate 5-mL cultures, which were allowed to incubate overnight at 37°C while shaking. Plasmids were then isolated (QIAprep Spin Miniprep Kit, Qiagen) from the One Shot Cells. Presence of the insert was determined by EcoR1 restriction digest, and plasmids containing the correct insert were sequenced using the T7 promoter primer at the University of California at Davis Sequencing Facility (ABI 3730 DNA Sequencer).

### cDNA and protein alignments

The feline cDNA sequence was obtained by aligning overlapping clones produced by the primer sets using CLUSTALW^a^. The feline GK protein sequence was derived by forward translation beginning from an open reading frame identified at the 3’ end of the assembled feline GCK cDNA. Comparative alignments of feline GCK cDNA and protein with sequences obtained from the respective NCBI databases were made with CLUSTALW. Nucleotide sequences used in comparative alignment were GenBank accessions human (NM_000162), chimpanzee (XM_001143661), rat (M25807), mouse (MN_010292). The protein sequences (with GenBank accessions) used were: human (NP_000153), chimpanzee (XP_001143661), rat (EDM00325); mouse (NP_034422).

### Molecular modeling

A structural model was generated for the feline glucokinase via homology modeling. To accomplish this, the target structure was predicted via alignment to a crystal structure of human GK (PDB ID: 1V4S) [11]. Initial sequence alignment for these two systems was performed with the Clustal-W program [22], using the Blosum 30 substitution matrix, a gap-opening penalty of 10 and a gap-extension penalty of 0.1. The crystal structure did not resolve the three residues closest to the N-terminus and four residues at the C-terminus, but over the remaining 448 positions, the mutual alignment yielded 95.5% identity and 98.4% similarity, which is indicative of an excellent template for comparative modeling. Based on this alignment and the corresponding three-dimensional human GK structure, we then predicted the structure of the analogous feline protein via the Modeller program [23], using default simulated annealing cycles for structural refinement.

## Endnote

^a^http://www.ebi.ac.uk/Tools/msa/clustalw2/ (link accessed 09/24/13).

## Abbreviations

GK: Glucokinase protein; GCK: Glucokinase gene; GKRP: Glucokinase regulatory protein; RACE: Rapid amplification of cDNA ends; ORF: Open reading frame; CDS: Coding DNA sequence; SNP: Single nucleotide polymorphism; UTR: Untranslated region; MODY: Maturity-onset diabetes in the young.

## Competing interests

Some of the results have been presented in a conference abstract: Vandersande, V., Lindbloom-Hawley, S., LeCluyse, M., Schermerhorn T., 2008. Journal of Veterinary Internal Medicine 22; 789–790 (Abstract 294). None of the authors has any financial or personal relationships that could inappropriately influence or bias the content of the paper.

## Authors’ contributions

SLH carried out the cloning studies and participated in cDNA and protein sequence alignments. ML participated in the cloning studies and the sequence alignments. VV participated in the cloning studies. GLH performed the molecular modelling and drafted the manuscript. TS conceived of the study, participated in its design and coordination, and drafted the manuscript. All authors read and approved the final manuscript.
